# Insights Into the Role of Extracellular DNA and Extracellular Proteins in Biofilm Formation of *Vibrio parahaemolyticus*

**DOI:** 10.3389/fmicb.2020.00813

**Published:** 2020-05-19

**Authors:** Wei Li, Jing Jing Wang, Hui Qian, Ling Tan, Zhaohuan Zhang, Haiquan Liu, Yingjie Pan, Yong Zhao

**Affiliations:** ^1^College of Food Science and Technology, Shanghai Ocean University, Shanghai, China; ^2^Laboratory of Quality and Safety Risk Assessment for Aquatic Products on Storage and Preservation, Ministry of Agriculture, Shanghai, China; ^3^Shanghai Engineering Research Center of Aquatic-Product Processing and Preservation, Shanghai, China; ^4^Engineering Research Center of Food Thermal-Processing Technology, Shanghai Ocean University, Shanghai, China

**Keywords:** *Vibrio parahaemolyticus*, biofilm, eDNA, extracellular proteins, EPS

## Abstract

The extracellular polymeric substances (EPS) construct the three-dimensional (3-D) structure of biofilms, but their respective roles are still not clear. Therefore, this study aimed to illuminate the role of key chemical components [extracellular DNA (eDNA), extracellular proteins, and carbohydrates] of EPS in biofilm formation of *Vibrio parahaemolyticus*. The correlations between each key chemical component and biofilm formation were first determined, showing that the biofilm formation of *V. parahaemolyticus* was strongly positively correlated with both eDNA and protein content (*P* < 0.01), but not with carbohydrates. Subsequently, individual DNase I or protease K treatment markedly reduced the initial adhesion and structural stability of the formed biofilms by hydrolyzing the eDNA or extracellular proteins, but did not induce significant dispersion of mature biofilms. However, the combination of DNase I and protease K treatment induced the obvious dispersion of the mature biofilms through the concurrent destruction of eDNA and extracellular proteins. The analysis at a structural level showed that the collapse of biofilms was mainly attributed to the great damage of the loop configuration of eDNA and the secondary structure of proteins caused by the enzyme treatment. Therefore, this study provides a deep understanding of the role of key chemical components of EPS in biofilm development of *V. parahaemolyticus*, which may give a new strategy to develop environmentally friendly methods to eradicate the biofilms in food industry.

## Introduction

*Vibrio parahaemolyticus* is recognized as a leading cause of seafood-derived food poisoning worldwide (Raszl et al., 2016), and it is ubiquitous in coastal waters or estuarine environments (Urmersbach et al., 2015; Mizan et al., 2016). *V. parahaemolyticus* has the high capacity to adhere to food-contact surfaces (aquaculture equipment, aquatic products, and food processing facilities) and forms biofilm (Han et al., 2016; da Rosa et al., 2018; Mougin et al., 2019). Biofilms are complex communities of microorganisms, which provides the encased microbial cells higher ability to tolerate environmental stresses such as antibiotics and disinfectants compared to planktonic cells (Costerton et al., 1999; Costa et al., 2013; Elexson et al., 2014; DeFrancesco et al., 2017). All these properties are attributed to bacterial cells embedded in firm three-dimensional (3D), multicellular, self-assembled structures that contain extracellular polymeric substances (EPS) (Costa et al., 2013; Flemming et al., 2016). EPS are the primary ingredient in bacterial biofilms, which typically accounts for greater than 90% dry mass of the biofilm (Brown et al., 2015). Of which, the key matrix components—DNA, proteins, and exopolysaccharides—are crucial for maintaining the structural integrity of biofilms providing a shelter for cells (Flemming and Wingender, 2010; Dragos and Kovacs, 2017).

Recent studies have shown that exopolysaccharides appear to be important for initiating and maintaining cell–cell interactions in biofilms, as well as protecting encased bacterial cells (Ophir and Gutnick, 1994; Colvin et al., 2011; Cugini et al., 2019). Proteins can provide 3D architectural integrity and surface adhesion for various bacterial biofilms, such as *Escherichia coli*, *Vibrio cholerae*, *Bacillus subtilis*, and *Vibrio vulnificus* (Hobley et al., 2015; Jin et al., 2016). More importantly, the groundbreaking discovery of extracellular DNA (eDNA) by Whitchurch et al. (2002) showed that eDNA is required for the initial establishment of *Pseudomonas aeruginosa* biofilms. Since this report, the roles of eDNA in biofilm formation, structural integrity, and tolerance to antibiotics have been widely described in other species (Okshevsky and Meyer, 2013; Rose et al., 2015; Ibanez de Aldecoa et al., 2017). Moreover, eDNA can be used as a source of nutrients for live cells and facilitate the spread of genetic traits in the biofilm and the planktonic populations (Chimileski et al., 2014; Brown et al., 2015). However, the chemical composition of EPS varies greatly depending on the bacterial species and the environment that the biofilm formed.

During the last decades, many studies mainly investigated the roles of exopolysaccharides and capsular polysaccharide (CPS) in the *V. parahaemolyticus* biofilm. Thereinto, Guvener and McCarter (2003) found that the mutants of *V. parahaemolyticus*, failing to produce CPS, formed defective biofilms. Nevertheless, Chen et al. (2010) proposed that the mutants mentioned above were related to exopolysaccharide production rather than CPS. Furthermore, they revealed the genes responsible for exopolysaccharide production in *V. parahaemolyticus* were located on chromosome II, that is, the VPA1403-1412 (*cpsA-J*) operon, whereas the loci VP0219-0237 in chromosome I was the capsule genes (K-antigen). Subsequently, Wang et al. (2013) showed that exopolysaccharides-deficient mutant of *V. parahaemolyticus* stained with much less crystal violet than wild type. Therefore, it can be concluded that the importance of exopolysaccharides and CPS in *V. parahaemolyticus* biofilm formation needs to be further clarified. Meanwhile, the exogenous addition of extracellular recombinant proteins significantly increased the biofilm formation of *V. parahaemolyticus* reaching ∼3.8-fold compared to control (Jung et al., 2019). However, the role of eDNA in the biofilm formation of *V. parahaemolyticus* is rarely reported. In addition, the role of chemical components of EPS in the biofilm formation of *V. parahaemolyticus* is still controversial.

In this study, *V. parahaemolyticus* was selected as a model organism to investigate the role of key chemical components (eDNA, extracellular proteins, and carbohydrates) of EPS in the biofilm development. To achieve this purpose, the dynamic process of biofilm formation was monitored by crystal violet staining, confocal laser scanning microscopy (CLSM) and scanning electron microscopy (SEM). The respective importance of chemical components of EPS in biofilm formation was revealed by Pearson correlation analysis and enzymatic hydrolysis treatment. Furthermore, the ISA-2 software analysis and Raman spectroscopy were used to characterize the structure changes of the biofilm treated by DNase I, proteinase K, and the combination of DNase I and proteinase K (DNase I–proteinase K). This study will reveal the role of chemical components of EPS in the biofilm formation of *V. parahaemolyticus* and hence provide effective strategy to design environmental-friendly, non-chemical methods to control biofilm formation in food industry.

## Materials and Methods

### Bacterial Strains and Cultivation

*Vibrio parahaemolyticus* ATCC17802 was used in this study and maintained in 50% (vol/vol) glycerol at −80°C. The single colony was inoculated in 9 mL tryptic soy broth (TSB; Beijing Land Bridge Technology Company Ltd., Beijing, China) supplemented with 3% (wt/vol) NaCl and incubated at 37°C for 12 h with shaking at 200 revolutions/min. After incubation, the broth culture was adjusted to OD_600_ = 0.4 corresponding to 4.1 × 10^7^ colony-forming units/mL, which was used for subsequent experiments.

### Biofilm Formation

Biofilm formation was performed according to the protocol previously described by Song et al. (2016) and Han et al. (2017). In detail, 24-well plates were filled with 990 μL of fresh TSB medium (3% NaCl) and then inoculated with 10 μL of the bacterial cultures (OD_600_ = 0.4). Then, the 24-well plates were incubated at 25°C statically to form biofilms under different times (2, 6, 12, 24, 36, and 48 h), and the wells containing TSB without inoculation were used as blank control. All plates were sealed with plastic self-sealing bags to prevent evaporation of water.

### Enzyme Treatment of Biofilms

Biofilms incubated at different times (2, 12, 24, 36, and 48 h) were washed once with 1 mL of 1 × phosphate-buffered saline (PBS). Subsequently, the biofilm samples were treated with DNase I (Roche), proteinase K (Sigma-Aldrich Co. LLC, Louis, United States), and the combination of DNase I and proteinase K at 37°C for 30 min. After static incubation, the wells were washed twice with 1 × PBS and stained with crystal violet to quantify the biofilm. For the experiments of enzyme treatment, all DNase I, proteinase K, and DNase I–proteinase K were used at a final concentration of 100 μg/mL unless otherwise stated. The biofilm without addition of enzymes was selected as control. All the experiments were repeated in at least three independent experiments.

### Crystal Violet Staining Assay

Biofilms of *V. parahaemolyticus* were quantified by crystal violet staining method (Crofts et al., 2018). Following static incubation, planktonic cells were removed from the wells before washing with 1 × PBS gently. After drying at 60°C for 15 min, the biofilms were stained with 1 mL of 0.1% (wt/vol) crystal violet (Sangon Biotech Co., Ltd., Shanghai, China) for 30 min at room temperature. The staining solution was removed via pipette, and then 1 × PBS was used to remove the non-bound dye at least three times. Stained and washed biofilms were air dried for 30 min, and then 1 mL of 95% ethanol was added to dissolve the bound crystal violet for 30 min. The optical density of each well was measured at wavelength of 600 nm using the BioTek Synergy 2 (Winooski, VT, United States).

### Visualization and Structural Analysis of the Biofilms Using Confocal Laser Scanning Microscopy

The *V. parahaemolyticus* biofilms were observed by CLSM. The biofilms on sterile glass were rinsed with 1 × PBS to remove loosely attached bacterial populations before fixed in 4% glutaraldehyde for 30 min at 4°C. Afterward, the staining solution of SYBR Green I (Sangon Biotech Co., Ltd.) was added to the well to completely submerge the glass, and then the biofilm was incubated for 30 min in the dark at room temperature. After that, all excessive staining solution was removed and air dried.

The confocal laser scanning microscope (TCS SP8; Leica Biosystems AG, Wetzlar, Germany) was employed to acquire biofilms images with 40 × objective. Excitation at 488 nm with an argon laser in combination with a 525 ± 25 nm band-pass emission filter was used for SYBR Green I signal visualization. Then, the volumetric parameters and textural parameters (biovolume, mean thickness, biofilm roughness, and porosity) were calculated from 3D CLSM images by the ISA-2 software to quantify the structural characteristics of *V. parahaemolyticus* biofilms (Beyenal et al., 2004a). The biovolume represents the overall volume of the biofilm in the observation field. Mean thickness provides a measure of the spatial size of the biofilm and is the common variable used in biofilm research. Roughness is calculated from the thickness distribution of the biofilm and gives a measurement of the variations in biofilm thickness and is an indicator of the superficial biofilm interface heterogeneity (Heydorn et al., 2000). Porosity is defined as the ratio of void area to total area (Beyenal et al., 2004b). For each sample, the image stacking was acquired with a 1-μm thickness at six random sites.

### Visualization of the Microstructure of the Biofilms Using Scanning Electron Microscopy

Biofilm samples formed on glass were washed by immersing in 1 mL of 1 × PBS and then mixed with 2.5% glutaraldehyde for overnight at 4°C (Tan et al., 2018; Chen et al., 2020). Subsequently, biofilm samples were dehydrated with increasing concentrations of ethanol at 30, 50, 70, and 90% for 10 min, respectively, followed by twice immersion in 100% ethanol for 10 min each (Liao et al., 2014). After air drying, biofilm samples were covered by using gold–palladium in an automatic sputter coater (Polaron SC7640 sputter coater; VG Microtech, East Sussex, United Kingdom) and visualized with the extreme-resolution analytical field emission scanning electron microscope (SM-7800F Prime; JEOL, Tokyo, Japan). The length of the biofilm cells was quantified by the ImageJ software (Rasband, W.S., ImageJ, US National Institutes of Health, Bethesda, MD, United States^1^, 1997–2014).

### Extraction and Chemical Components Analysis of EPS

The EPS of *V. parahaemolyticus* biofilms were extracted using the sonication method (DeFrancesco et al., 2017; Tan et al., 2018). Briefly, the medium was aspirated, and the remaining adherent cells were washed with sterile PBS. Biofilms were then resuspended in 1 mL 0.01M KCl solution and collected by vortexing and scraping. Next, *V. parahaemolyticus* biofilm cell clumps were dispersed with a Scientz-IID sonicator (Ningbo Scientz, Ningbo, Zhejiang, China) for six cycles of 5 s of operation and 5 s of pause at a power level of 20k Hz (45w) (Ding et al., 2019; Chen et al., 2020). The sonicated suspension was pelleted by centrifugation for 10 min at 7000 × *g* at 4°C, and then the supernatant was removed and filtered by using a 0.22-mm membrane filter (Sangon Biotech Co., Ltd.). The amounts of DNA, proteins, and carbohydrates in the filtrate were analyzed. DNA was detected by using the Quant-iT^TM^ PicoGreen^®^ dsDNA Assay Kit (Invitrogen^TM^ Ltd., Paisley, United Kingdom) according to manufacturer’s instructions (Grande et al., 2015). The protein content was determined using Lowry method by Stable Lowry Protein Assay Kit (Sangon Biotech Co., Ltd.). The concentration of carbohydrate was quantified by the phenol–sulfuric acid method using glucose as a stand (Kim and Park, 2013). After that, the correlation analysis was created based on the OD_600_ value versus the content of three chemical components after the biofilm was incubated at different times (2, 6, 12, 24, 36, and 48 h). Meanwhile, the correlation analysis between the contents of three different chemical components was also performed. Per experiment was tested in at least three replicates.

### Raman Spectroscope Analysis

The EPS of mature biofilms after DNase I, proteinase K, and DNase I–proteinase K treatment were extracted as described in the extraction and chemical component analysis of EPS. Additionally, the EPS of mature biofilms without enzyme treatment were used as control. The Raman spectra of four EPS samples were recorded with a Senterra R200-L Dispersive Raman Microscope (Bruker Optics, Ettlingen, Germany) at room temperature. A diode laser at 633 nm and 50 × objective with a laser power of 3 mW was used for all Raman experiments. The Raman spectrum of each sample was calculated as the average of five measurements at different arbitrary sites on the biofilm. All Raman measurements were recorded with an accumulation time of 60 s in the range of 400–1520 cm^–1^. The acquisition of Raman spectrum and preprocessing of preliminary data were conducted using the Bruker OPUS software.

### Statistical Analysis

The experimental data were expressed as the mean ± standard deviation. Statistical analysis was carried out by one-way analysis of variance using SPSS version 21.0 (SPSS Inc., Chicago, IL, United States) to compare the value differences (*P* < 0.05).

## Results

### The Dynamic Process of Biofilm Formation

**FIGURE 1:**
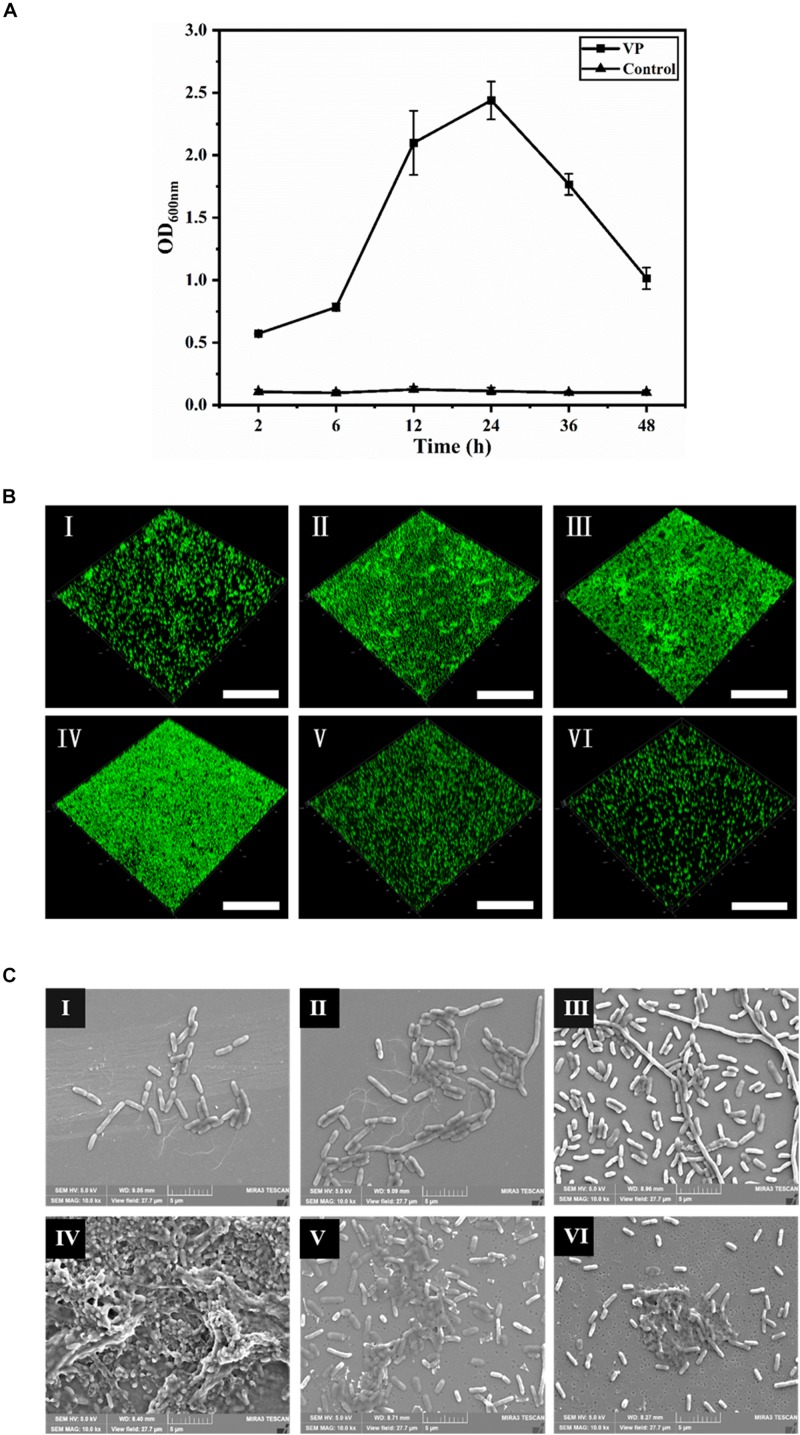
The dynamic process of *V. parahaemolyticus* biofilm formation. **(A)** Biofilm biomass was measured by crystal violet binding at an OD_600_ after static culture for different times (2, 6, 12, 24, 36, and 48 h). Error bars show standard deviations of three independent experiments. **(B)** Confocal laser scanning microscopic images of *V. parahaemolyticus* biofilm development process. The scale bar represents 100 μm. **(C)** Scanning electron microscopic images of microstructure during the development of *V. parahaemolyticus* biofilm. The scale bar represents 5 μm. I–VI represent 2, 6, 12, 24, 36, and 48 h, respectively. Pictures are representative of three independent experiments with at least three replicates each.

The dynamic process of *V. parahaemolyticus* biofilm formation was monitored in terms of the absorbance of the dissolved crystal violet dye from microtiter plates (Figure 1A). In the initial attachment phase (2–6 h), the biofilm formation was not obvious, and then a rapid increase in biofilm formation was tested in 6–12 h. After 12 h, the development of biofilms slowed down gradually. Subsequently, the biofilms further developed and reached the maximum at 24 h with an OD_600_ of 2.44. After 24 h, the biofilms entered the dispersion stage leading to a sharp decrease of biomass.

The changes of *V. parahaemolyticus* biofilms were visualized using CLSM (Figure 1B), and their structural parameters were quantified in Table 1. Almost all cells were distributed as a single cell at 2 h, and then the biofilm cells gradually increased and formed scattered aggregates from 2 to 6 h, indicating the ongoing colonization of bacterial cells on the surfaces. At 6 h, the surface was covered by a slightly dense biofilm, with its biovolume and mean thickness being only 4.38 × 10^5^ μm^3^ and 2.47 μm. When the time was prolonged to 12 h, the cells formed large clusters; furthermore, the architectures of biofilm developed from a single-layer planar structure to a multilayer 3D structure. Accordingly, the biovolume and mean thickness of the biofilm significantly increased to 6.57 × 10^5^ μm^3^ and 5.32 μm. Conversely, the biofilm roughness and porosity were greatly reduced to 0.96 and 0.82, respectively. Mature biofilm with minimum roughness and porosity (0.89 and 0.77) was detected at 24 h. Meanwhile, the biovolume and mean thickness of biofilm reached the maximum, values of 9.36 × 10^5^ μm^3^ and 8.44 μm, respectively. After 36 h of cultivation, the biofilm appeared disaggregated morphology where its biovolume and mean thickness decreased to 5.24 × 10^5^ μm^3^ and 4.10 μm. After 48 h, further disaggregation of the biofilm led to a reduction in the biovolume and mean thickness, indicating the low concentration of microorganisms on the surface. Therefore, the biofilm roughness and porosity markedly increased compared to mature biofilm.

**TABLE 1:** Quantification of the biofilm structure of *V. parahaemolyticus* after 2, 6, 12, 24, 36, and 48 h of incubation.

Structural parameters	Time points (h)					
	2	6	12	24	36	48
Biovolume × 10^5^ (μm^3^)	3.39 ± 0.80	4.38 ± 0.46	6.57 ± 0.26	9.36 ± 0.85	5.24 ± 0.68	3.73 ± 0.38
Mean thickness (μm)	1.55 ± 0.37	2.47 ± 0.34	5.32 ± 0.37	8.44 ± 0.75	4.10 ± 0.27	3.59 ± 0.50
Biofilm roughness	1.49 ± 0.09	1.21 ± 0.10	0.96 ± 0.06	0.89 ± 0.12	1.26 ± 0.10	1.27 ± 0.09
Porosity	0.92 ± 0.01	0.86 ± 0.01	0.82 ± 0.01	0.77 ± 0.01	0.87 ± 0.01	0.92 ± 0.02

Scanning electron microscopy was used to observe the changes of microstructures during the biofilm development (Figure 1C), and the length of biofilm cells is shown in Table 2. In the initial adhesion stage, the biofilm was mainly composed of individual cells, and the morphology of cells became filamentous shape to better colonize on the contact surfaces. Simultaneously, the mean length and maximum length of biofilm cells increased to 2.64 and 15.50 μm at 6 h, respectively. The cells further elongated themselves toward the center of microcommunity to form large aggregates after 12-h incubation. Likewise, the maximum length exhibited a great increase ranging from 15.50 to 34.23 μm, and the mean length decreased slightly from 2.64 to 2.36 μm. The mature biofilm with dense and complex 3D structures was acquired at 24 h, and most of the cells adhered closely and were held together by EPS, and therefore, the mean length and maximum length were not measurable. At 36 h, the 3D structures of the *V. parahaemolyticus* biofilms dissipated releasing individual cells. Obviously, the mean length and maximum length of biofilm cells decreased dramatically to 2.01 and 6.81 μm. The dynamic processes of biofilm development characterized by CLSM and SEM were consistent with the results of crystal violet staining.

**TABLE 2:** Quantification of the length of biofilm cells of *V. parahaemolyticus* after 2, 6, 12, 24, 36, and 48 h of incubation.

Cell length (μm)	Time points (h)					
	2	6	12	24	36	48
Mean length	2.05 ± 0.62	2.64 ± 2.14	2.36 ± 4.27	Not measurable	2.01 ± 0.76	1.59 ± 0.45
Maximum length	4.48	15.50	34.23	Not measurable	6.81	2.77

### Correlation Between EPS and *Vibrio parahaemolyticus* Biofilm Formation

**FIGURE 2:**
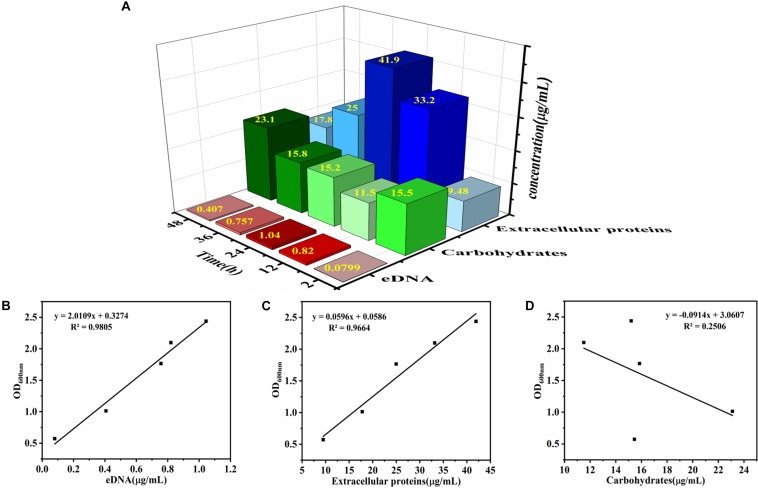
Determination of chemical component contents in EPS and the correlation analysis between chemical component contents and *V. parahaemolyticus* biofilm formation. **(A)** The contents of eDNA, extracellular proteins, and carbohydrates in EPS were determined after 2, 6, 12, 24, 36, and 48 h of cultivation. The color of red, green, and blue represent eDNA, carbohydrates, and extracellular proteins, respectively. The intensity of the color represents the level of chemical component contents in EPS. Correlation analysis between chemical component contents in EPS and biofilm formation: **(B)** eDNA, **(C)** extracellular proteins, and **(D)** carbohydrates.

**FIGURE 3:**
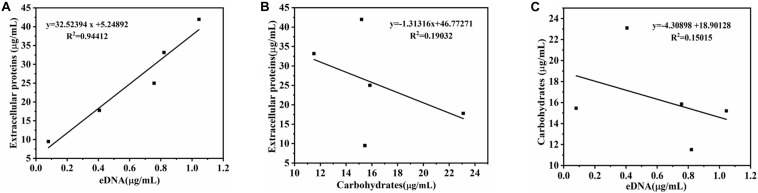
Correlation analysis between eDNA, extracellular proteins, and carbohydrates of EPS in biofilm development of *V. parahaemolyticus*. **(A)** eDNA and extracellular proteins, **(B)** eDNA and carbohydrates, and **(C)** carbohydrates and extracellular proteins.

The relationship between biofilm formation and EPS was investigated using Pearson correlation analysis. The contents of eDNA, extracellular proteins, and carbohydrates were determined in Figure 2A. The results showed that the extracellular proteins and carbohydrates were the main components of EPS of the mature biofilm, followed by eDNA, which accounted for 72, 26, and 2% by mass, respectively. Interestingly, the three major components in EPS showed different changes during biofilm development. Of which, the amount of eDNA and extracellular proteins presented positively linear correlation with the biofilm formation, and the corresponding Pearson correlation coefficients (*r*_p_) reached 0.99 (*R*^2^ = 0.9805) and 0.983 (*R*^2^ = 0.9664), respectively. However, no linear correlation was observed for carbohydrate content and biofilm formation (Figures 2B–D). Meanwhile, there was a strong positive correlation between eDNA and extracellular proteins (*r*_p_ = 0.972, *P* < 0.01) (Figure 3A). However, there was no correlation between carbohydrates and eDNA (*r*_p_ = -0.387, *P* > 0.05), as well as extracellular proteins (*r*_p_ = -0.436, *P* > 0.05) (Figures 3B,C). These results suggested that there should be collaborative functions of eDNA and extracellular proteins on the biofilm formation of *V. parahaemolyticus.*

### Effect of eDNA and Extracellular Proteins on Biofilm Formation of *V. parahaemolyticus*

**FIGURE 4:**
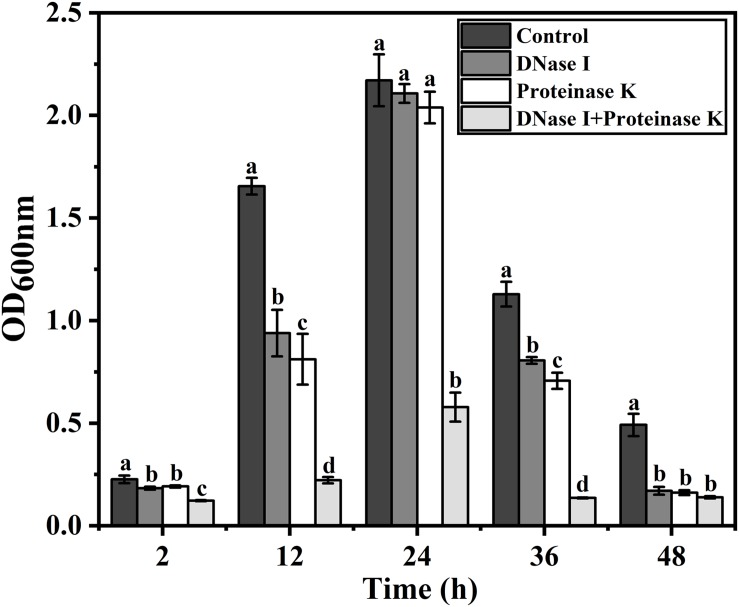
Effect of eDNA and extracellular proteins on biofilm formation of *V. parahaemolyticus*. To confirm whether the eDNA and extracellular proteins serve as structural components in biofilms of *V. parahaemolyticus*, DNase I, proteinase K, and their combination were added to the biofilms incubated at different times (2, 12, 24, 36, and 48 h). Error bars show standard deviations of three independent experiments, and the different letters represent significant differences among treatments (*P* < 0.05).

To determine the role of eDNA and extracellular proteins in biofilm formation of *V. parahaemolyticus*, DNase I, proteinase K, and their combination were added to the biofilms incubated at different times (2, 12, 24, 36, and 48 h). The amount of biofilm after DNase I, proteinase K, and DNase I–proteinase K treatment was determined by crystal violet staining (Figure 4), and their eradication efficiency is listed in Table 3. At the early stage (2 and 12 h), the addition of DNase I or proteinase K greatly decreased the initial attachment and destroyed the stability of formed biofilms (*P* < 0.05), proving that the eDNA and extracellular proteins were important for both initial attachment of cells and subsequent biofilm development. However, the 24-h-incubated biofilms with dense 3D structures were more resistant to individual DNase I and proteinase K treatment. However, great damage of biofilm treated by the combination of DNase I and proteinase K was obtained during all stages compared to individual enzyme treatment. Therefore, the combination of DNase I and proteinase K could effectively remove the mature biofilms, resulting in a reduction of 62.86% of the biomass.

**TABLE 3:** Clearance of biofilm by different enzymes (%).

Enzyme treatment (30 min)	Time points (h)				
	2	12	24	36	48
DNase I	21.49^i^ ± 1.82	46.35^f^ ± 1.37	6.87^j^ ± 0.23	27.46^g^ ± 0.80	66.85^c^ ± 1.99
Proteinase K	18.11^i^ ± 0.54	50.98^e^ ± 2.38	6.04^j^ ± 0.21	37.41^h^ ± 0.43	65.64^cd^ ± 3.34
DNase I + proteinase K	46.70^f^ ± 3.82	88.69^a^ ± 2.38	62.86^d^ ± 2.6	87.78^a^ ± 0.52	71.57^b^ ± 2.78

*The different letters represent significant differences among treatments (P < 0.05).*

### Effects of eDNA and Extracellular Proteins on Architectures of the *V. parahaemolyticus* Biofilms

**FIGURE 5:**
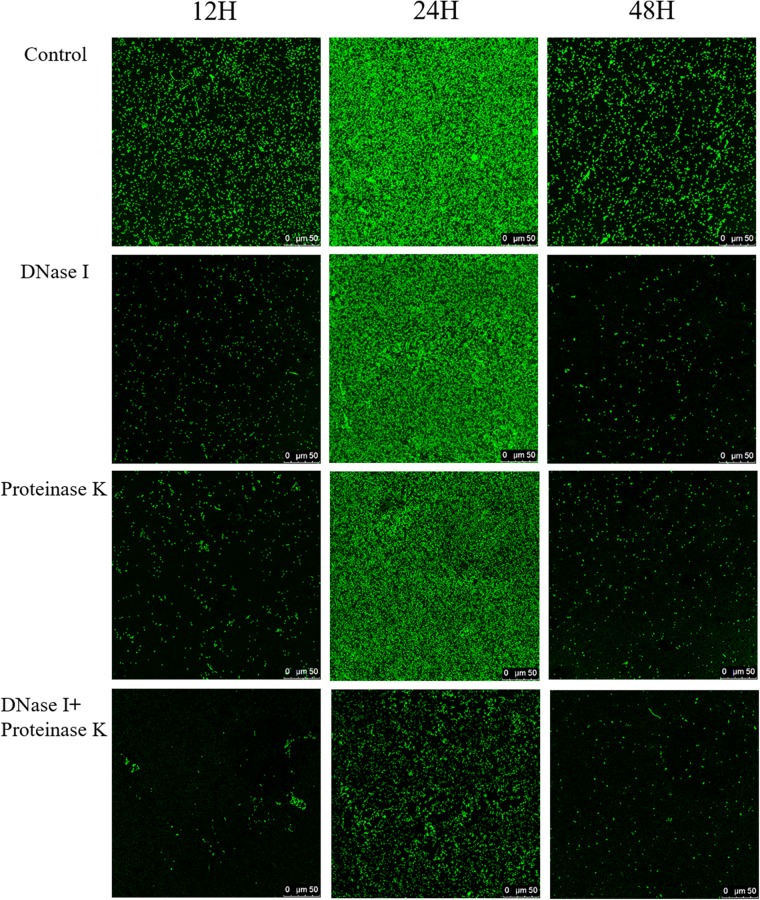
Representative CLSM images of *V. parahaemolyticus* biofilms treated with DNase I, proteinase K, and their combination at 12, 24, and 48 h of cultivation. The scale bar represents 50 μm. Pictures are representative of at least three individual scans from three independent experiments.

**FIGURE 6:**
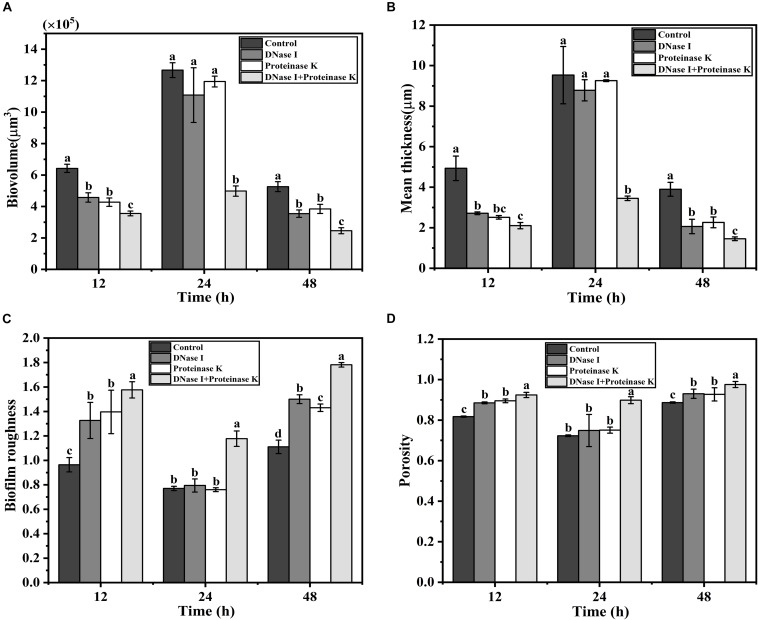
Structural characteristics changes of *V. parahaemolyticus* biofilms treated with DNase I, proteinase K, and their combination at 12, 24, and 48 h of cultivation. **(A)** Biovolume, **(B)** mean thickness, **(C)** biofilm roughness, and **(D)** porosity. Error bars show standard deviations of three independent experiments, and the different letters represent significant differences among treatments (*P* < 0.05).

Confocal laser scanning microscopy was employed to verify the effects of eDNA and extracellular proteins on the architectures of *V. parahaemolyticus* biofilms in combination with ISA-2 software. The morphology differences of treated and untreated biofilms in development (12 h), maturation (24 h), and dispersion stage (48 h) are shown in Figures 5, 6. The biofilms in development stage (12 h) were corroded and showed irregular black holes after individual DNase I or proteinase K treatment. More sparse and decreased biofilms were observed after DNase I–proteinase K treatment. Likewise, the 48-h-incubated biofilms almost disappeared after DNase I, proteinase K, and DNase I–proteinase K treatment. However, there were no significant differences in the morphology of mature biofilms treated by individual enzyme compared to control samples. However, the DNase I–proteinase K-treated biofilms presented the markedly reduced amount and unevenly dispersed structures. Quantitative analysis revealed that the biovolume of biofilms was 6.4 × 10^5^ μm^3^ in control samples (Figure 6A). After treatment of DNase I, proteinase K, and DNase I–proteinase K, the biovolume of 12-h-incubated biofilms was highly decreased to 4.6 × 10^5^, 4.3 × 10^5^, and 3.6 × 10^5^ μm^3^, respectively. However, the individual DNase I or proteinase K lost the ability to destroy the mature biofilms (24 h), although these DNase I–proteinase K could still greatly decrease the biomass of mature biofilms. When the biofilms entered the dispersion stage, the changes in the structural parameters were similar to those of 12-h-incubated samples under different enzyme treatment. Furthermore, the biofilm thickness of 12-h-incubated biofilms treated with different enzymes exhibited a great (*P* < 0.05) decrease ranging from 5.0 to 2.1 μm (Figure 6B). Such similar changes also occurred in 48-h-incubated biofilms. However, only the mature biofilms (24 h) treated by DNase I–proteinase K showed a reduction of 6-μm thickness and disappearance of multilayer structures. In Figure 6C, the biofilm roughness values of 12-h-incubated biofilms markedly (*P* < 0.05) increased from 0.96 to 1.58 after different enzyme treatment. When the biofilms entered the maturation stage, the mature biofilms presented intensively distributed architectures, which effectively prevented the actions of the individual DNase I or proteinase K. Nonetheless, the mature biofilms treated with DNase I–proteinase K showed a higher biofilm roughness (1.18 > 0.77) than the control samples. For the 48-h-incubated samples, the changes in their roughness were similar to those of 12-h-incubated samples. In addition, the porosity significantly (*P* < 0.05) increased in both 12- and 48 h-incubated biofilms treated by different enzymes (Figure 6D). For mature biofilms (24 h), their porosity did not present significant change after individual enzyme treatment. However, the porosity obviously increased from 0.72 μm in control samples to 0.90 μm in DNase I–proteinase K-treated samples. All the results suggested that eDNA and extracellular proteins were the key structural components of biofilm maintaining the mechanical stability of the mature *V. parahaemolyticus* biofilms.

### Raman Analysis of Enzyme-Treated Mature Biofilms of *Vibrio parahaemolyticus*

**FIGURE 7:**
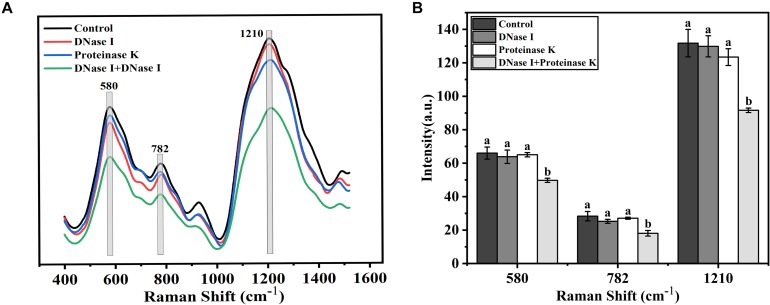
Raman spectrum and intensity changes of mature biofilms of *V. parahaemolyticus* after treatment with DNase I, proteinase K, and their combination. **(A)** Raman spectrum and **(B)** intensity changes. Error bars show standard deviations of three independent experiments with five measurements each, and different letters represent significant differences among treatments (*P* < 0.05).

Raman spectrum was used to monitor the chemical structure changes of eDNA and extracellular proteins in mature biofilms treated by different enzymes. The representative Raman spectra of eDNA and extracellular proteins are shown in Figure 7, and the tentative peak assignments are summarized in Table 4. The typical bands of 561 to 582 cm^–1^ could be assigned to glycosidic ring deformation vibration (COC) in carbohydrates. In this region, the band intensity of the EPS were dramatically weakened after DNase I–proteinase K treatment. Similar change trends were also observed in the region of 780–788 cm^–1^ (O-P-O stretching; cytosine, uracil) corresponding to DNA. Amide III (1200–1300 cm^–1^, associated with C-N stretching and N-H bending) bands were the indicator of the secondary structure of proteins. No significant change in the peak intensity of proteins was observed after individual DNase I or proteinase K treatment, but the intensity was obviously decreased after DNase I–proteinase K treatment, indicating that the enzymes induced the changes of secondary structure of proteins.

**TABLE 4:** Peak assignment for Raman spectra of EPS in biofilms.

Peak position (cm^–1^)	Assignment	Macromolecular assignment	References
561–582	C-O-C glycosidic ring def; COO- wag; C-C skeletal	Carbohydrates	Guicheteau et al., 2008; Ivleva et al., 2008; Han et al., 2017
780–788	O-P-O str; cytosine (C), uracil (U)	Nucleic acids	Ivleva et al., 2009; Samek et al., 2010; Liu et al., 2014; Ramirez-Mora et al., 2019
1200–1300	Amide III	Proteins	Notingher, 2007; Samek et al., 2014

*def, deformation vibration; str, stretching.*

## Discussion

*Vibrio parahaemolyticus* has been considered as the main human pathogen in marine bacteria because of the increasing number of outbreaks and infections and the ability to form biofilms easily on various surfaces (Martinez-Urtaza et al., 2013; Mizan et al., 2016). Biofilm formation is a serious problem in food industries where the biofilms can be a persistent source of contamination (Van Houdt and Michiels, 2010). Biofilm development is a complex physiological process guided by a series of physical, chemical, and biological factors, and the importance of EPS are well established for biofilm formation (Flemming et al., 2016; Dragos and Kovacs, 2017; Koo et al., 2017). Although EPS provide structural support to maintain the biofilm stability of *V. parahaemolyticus*, their respective functional role is still unclear (Han et al., 2017; Tan et al., 2018

The production of high amount of EPS and the formation of densely distributed architectures were the predominant characteristics of the mature biofilms (Gupta et al., 2015; Zhang et al., 2015). After 24 h incubation, the biofilms with 3D structures and multilayers formed (Figure 1B). Moreover, a large amount of EPS secreted by *V. parahaemolyticus* was observed (Figure 1C). All these results indicated that the biofilm formation of *V. parahaemolyticus* reached maturation stage after 24-h incubation. Tan et al. (2018) reported that the biofilm formation of *V. parahaemolyticus* VPS36 entered the maturation stage after 48-h incubation, in which the mature cycle was twice the fold of that of *V. parahaemolyticus* ATCC17802. Such significant difference was collectively contributed by the following factors including the bacterial species, the type of attachment surfaces, and the environmental conditions (pH, temperature, nutritional conditions) (Whitehead and Verran, 2015). Among these factors, the bacterial species appears to act a pivotal role in biofilm formation owing to the heterogeneity of *Vibrio* strains (Song et al., 2016; Odeyemi and Ahmad, 2017; Mougin et al., 2019).

Once a mature biofilm community is established, the resistance to the harsh environments will be strengthened due to the functions of EPS (Nilsson et al., 2011). It is now widely accepted that three chemical components of the EPS, namely, eDNA, proteins, and exopolysaccharides, are assigned to specific structural roles in biofilm formation (Dragos and Kovacs, 2017). The quantitative analysis of the EPS from *V. parahaemolyticus* showed that the extracellular proteins and carbohydrates were the main components of EPS of the mature biofilm, followed by eDNA (Figure 2). Furthermore, the amount of eDNA and extracellular proteins was highly correlated with the biofilm formation of *V. parahaemolyticus*, but the carbohydrate content showed no correlation (Figure 2). Meanwhile, a significant correlation (*r*_p_ = 0.972, *P* < 0.01) was observed between eDNA and extracellular proteins (Figure 3). All these facts suggested that the eDNA and extracellular proteins acted a leading role in biofilm formation. Such results were consistent with previous studies. For example, eDNA and DNABII proteins were central to the overall architecture and structural integrity of the non-typable *Haemophilus influenzae* biofilms (Goodman et al., 2011; Jurcisek et al., 2017). Additionally, eDNA acted as an electrostatic net to interconnect cells surrounded by positively charged matrix proteins in the *Staphylococcus aureus* biofilms (Dengler et al., 2015). Likewise, Huseby et al. (2010) found that eDNA-medicated cross-linking of β toxin facilitated the formation of the skeletal framework during the biofilm development of *Staphylococcus*. However, in this study, the amount of carbohydrates did not seem to have an effect on biofilm formation, despite its high content. Similar results were obtained by Ding et al. (2019), who reported that the polysaccharide content showed no correlation (*r*_p_ = 0.61, *P* > 0.05) to biofilm growth. In addition, the group demonstrated that proteins rather than polysaccharides in the EPS from strain XL-2 played the dominant role in biofilm formation. In *Helicobacter pylori*, eDNA may not be the main component of biofilm matrix, but studies have shown that eDNA played an important role in biofilm formation by “bridging” OMV–OMV (outer membrane vesicles) and OMV–cell interactions (Grande et al., 2011, 2015). Hence, we speculated that the above phenomenon was due to that the components of EPS exhibited different roles in the biofilm formed by different bacteria.

Extracellular DNA has been reported to possess a significant effect on the initial attachment and stability of biofilm structure in Gram-positive and Gram-negative bacteria (Das et al., 2013; Okshevsky et al., 2015). In this study, the DNase I treatment induced different degrees of damage to the biofilm (Figure 4). In particular, the addition of DNase I significantly decreased the amount of eDNA at early stage (2 and 12 h) leading to an obvious collapse of biofilms (Figure 4), which indicated that eDNA was crucial for initial attachment and development of biofilms. Previously, Godeke et al. (2011) applied DNase I to investigate the functions of eDNA in the biofilm formation; their results suggested that eDNA enhanced the initial surface attachment of bacterial cells and was the key structural component in all stages of *Shewanella oneidensis* biofilm formation. Meanwhile, Harmsen et al. (2010) found that eDNA played an essential role in the attachment and development of the *Listeria monocytogenes* biofilms. In addition, Das et al. (2010, 2011) revealed that eDNA contributed to the bacterial adhesion and aggregation mainly by the attractive Lifshitz–van der Waals and acid–base interactions.

Extracellular proteins, the main component of EPS, were proved to be an indispensable functional component for biofilm formation of *V. parahaemolyticus*. It was reported that extracellular proteins were structural elements of biofilm and exerted important functions during biofilm formation (Karunakaran and Biggs, 2011). Additionally, the present results were also supported by both Kumar Shukla and Rao (2013) and Ding et al. (2019), finding that the biofilm hydrolyzed with proteinase K showed a significant decrease in biomass. However, individual protease K treatment did not induce the dispersion of mature biofilm. Similar results were also observed for DNase I treatment (Figure 4). Notably, DNase I–protease K treatment induced the dispersion of 62.86% biofilm compared to control samples (Table 3). Therefore, the eDNA and extracellular proteins collectively played a critical role in the development and structure integrity of the *V. parahaemolyticus* biofilm. Moreover, Lappann et al. (2010) found that the crude chromosomal DNA could readily promote the biofilm formation and structure of *Neisseria meningitidis*, whereas pure DNA or DNase I-treated or proteinase K-treated crude DNA lost the improvement ability. The above phenomenon suggested that there should be a coaction of DNA and proteinaceous constituents, which promoted the biofilm formation and mechanical stability.

Structural parameters analysis showed that the density of biofilm cells decreased and the porosity of biofilm increased after DNase I–protease K treatment, indicating that the biomass of biofilm was decreased and the structure stability was destroyed (Figures 5, 6). The results of Raman spectrum also supported above facts. Compared with individual enzyme treatment, the DNase I–protease K treatment destroyed the loop configuration of eDNA and the secondary structure of proteins, which caused the collapse of EPS leading to the dispersion of biofilm (Figure 7). A similar finding was reported by Jung et al. (2014), who observed that the destruction of DNA ring structure and conformational changes of proteins decreased DNA and proteins when the biofilms were treated with antibiotics. In addition, Chen et al. (2020) revealed the degradation of DNA (guanine, cytosine, and uracil) and proteins (amide III phenylalanine), or altering the conformation of functional groups destroyed the chemical composition of EPS under photodynamic inactivation treatment. Previously, Das et al. (2011) proposed that when the loop configuration of the eDNA in *Streptococcus* was changed into a more trainlike configuration, no specific adsorption sites were available for cross-linking with other bacteria on the cell surface, which led to the decrease of aggregation. Moreover, Liu et al. (2008) and Das et al. (2011) also found that the loop configuration of the eDNA played a vital role in biofilm formation and aggregation. In addition, the key role of protein secondary structures in promoting adhesion, aggregation, and biofilm formation has been widely reported (Wang et al., 2018; Ding et al., 2019). Although this study has clarified that eDNA and extracellular proteins collectively contributed to the mature biofilm formation of *V. parahaemolyticus*, the interactions between these two components are needed to be further investigated. Swinger and Rice (2004) reported that IHF (DNABII proteins) binds DNA, depending on significant sequence specificity. Additionally, Kavanaugh et al. (2019) suggested that extracellular proteins facilitated *S. aureus* biofilm formation by linking individual bacterial cells together through non-covalent cross-links with eDNA.

## Conclusion

The EPS, mainly composed of eDNA, extracellular proteins, exopolysaccharides, and so on, directly mediate the adhesion of microorganisms to surfaces to develop the complex 3D structure of biofilms. In this study, the amount of eDNA and extracellular proteins was positively correlated with the biofilm formation of *V. parahaemolyticus*, but the carbohydrates showed no correlation. The destruction of the eDNA or extracellular proteins greatly decreased the attachment and stability of the formed biofilms in early stage, but did not produce obvious destruction on mature biofilms. However, the concurrent destruction of the eDNA and extracellular proteins induced the dispersion of the mature biofilms after DNase I–protease K treatment. Further analysis showed that the collapse of biofilms was mainly attributed to the damage of the loop configuration of eDNA and the secondary structure of proteins caused by the enzymes. Therefore, the illumination of the role of chemical components in EPS may provide a further understanding of biofilm formation mechanisms of *V. parahaemolyticus* and also give novel insight to establish environmentally friendly cleaning methods to eliminate the biofilms in food industry.

## Data Availability Statement

All datasets generated for this study are included in this manuscript.

## Author Contributions

YZ, JW, YP, and HL conceived and supervised the study. WL and HQ designed the experiments. WL performed the experiments, analyzed the data, and wrote the manuscript. JW, LT, ZZ, and YZ revised the manuscript.

## Conflict of Interest

The authors declare that the research was conducted in the absence of any commercial or financial relationships that could be construed as a potential conflict of interest.

## Footnotes

1 http://imagej.nih.gov/ij/
